# Integration of the arts and biomedical engineering in innovative interdisciplinary anatomy education: The synthetic anatomy module

**DOI:** 10.1002/ase.70285

**Published:** 2026-06-11

**Authors:** Mandeep Gill Sagoo, Pak Yin Lam, Leigh Wilson, Les Bicknell, Kawal Rhode, Siham Omar, Richard Wingate

**Affiliations:** ^1^ Faculty of Life Sciences and Medicine King's College London London UK; ^2^ Institute of Psychiatry, Psychology & Neuroscience King's College London London UK; ^3^ Norwich University of the Arts Norwich UK

**Keywords:** 3D printing, anatomical modelling, anatomy education, arts‐based antomy learning, biomedical engineering education, constructivist learning, creative pedagogy, experiential learning, interdisciplinary learning, reflective practice, speculative design, STEM education, student‐centred learning, Synthetic anatomy, visual storytelling

## Abstract

The intersection of science and art has historically fostered innovation, yet the integration of creative arts into anatomy education remains limited. The Synthetic Anatomy module was designed to bridge anatomy teaching with creative design for bioscience and biomedical engineering students. This study evaluates the module's educational impact and explores student perceptions of integrating artistic approaches into anatomy learning. Over 10 weeks, student groups conceptualize and 3D‐print anatomical structures that transcend clinical and evolutionary norms. This is supplemented with workshops led by visiting artists and PhD biomedical engineering students. Each group presents an unmanned table display of models on a self‐selected topic, including bionic arms, human wings, and hybrid human‐avian hearts. Feedback was collected from 89 students and six tutors across three academic years (2018–2020), through focus groups and semi‐structured interviews respectively. Qualitative responses were analyzed using inductive thematic analysis. The analysis revealed three key themes: creative exploration, experiential learning, and interdisciplinary collaboration. Students valued the freedom in exploring imaginative anatomical designs beyond textbook conventions. The unmanned displays encouraged inventive presentation methods using motion, sound, and touch. The module's wider impact was illustrated by a 2017 biomedical engineering student who further developed prosthetic chest‐wall design for cancer patients, later published in *Frontiers of Surgery* and featured on BBC *Click*. The module has supported student‐led design concepts that extend anatomy learning into creative and translational contexts. Ongoing improvements are being made through student feedback and pedagogical reflection, alongside efforts to measure long‐term educational impact.

## INTRODUCTION

The intersection between the sciences and arts has traditionally been a fertile ground for innovation. However, recent educational trends have distanced creativity from scientific disciplines, particularly fields such as anatomy.^1^ Creativity, as defined by Colman,^2^ involves the production of ideas and objects that are both novel and meaningful. Despite the natural synergy between anatomy and the creative arts, previous work has highlighted the potiential value of art‐based approaches in medical education, particularly for developing observational skills,^3^ while creative projects in anatomy have also been shown to support reflection on professionalsm and may help some students amnage stress.^4^ However, contemporary anatomy curricula have often been dominated by structured factual learning and assessment‐driven outcomes; as a result, opportunities for students to engage creatively with anatomical knowledge or explore speculative design applications remain limited.^1^, ^5^, ^6^ This reduction in creative engagement may constrain students' ability to apply anatomical understanding in innovative or interdisciplinary contexts, particularly in emerging fields such as biomedical engineering, medical design, and patient‐specific technologies. This decline is particularly concerning in light of educational theories such as Piaget's constructivism^7^ and Vygotsky's social constructivism,^8^ which emphasize active, socially mediated and creative forms of learning.

In response to these limitations, the Synthetic Anatomy (SA) module was developed in 2017 to explore how creative practice and speculative design could be integrated into anatomy education. The module brings together bioscience (anatomy) and biomedical engineering students to engage with anatomical knowledge through artistic experimentation, collaborative design, and digital fabrication techniques. This dual emphasis on creative thinking and technical skill‐building positions it as a forward‐looking model in STEM education. The module draws on key pedagogical models such as Kolb's experiential learning cycle,^9^ Biggs' constructive alignment,^10^ and Bloom's taxonomy^11^ to ensure that creative exploration is structured, meaningful, and academically rigorous.

The module has two distinct parts: (1) an introduction to computer modeling and three‐dimensional (3D)‐printing techniques, followed by (2) an application of these skills in a highly supported but self‐directed project. The second part is scaffolded by a series of workshops on lateral thinking (approaching a problem from a new or non‐traditional angle), creative problem‐solving, and artistic critique, which inspire and guide student‐led designs. This can range from futuristic adaptations of the human body to the developmental reconstructions of extinct species or mythical creatures. The integration of creative exploration aims to highlight the importance of creativity as a vital component of learning, challenging the conventional boundaries of anatomy education with a greater focus on exploring the dynamic nature of anatomy, and developing a more holistic understanding of the human body.

The module's design is rooted in several key educational theories that emphasize experiential, collaborative and creative learning. Cognitive constructivism, proposed by Piaget, underpins the module's approach to learning through experience and adaptation to new challenges. Social constructivism, influenced by Vygotsky, is evident in the emphasis on group work and peer interaction, where learning is scaffolded through social engagement. Kolb's experiential learning theory is reflected through hands‐on activities such as 3D‐printing and creative arts sessions, where students learn by doing, reflecting and applying their knowledge in novel ways. Biggs' constructive alignment theory forms the basis of the module's alignment of learning objectives, teaching activities and assessments, ensuring that all aspects of the course work together to promote deep learning. Bloom's taxonomy underpins the formulation of learning outcomes that encourage higher‐order thinking, particularly through the creative and critical application of anatomical knowledge in student project design.

The module encourages students to explore speculative design ideas and test unconventional anatomical concepts, promoting an educational environment where unsuccessful prototypes or design attempts are treated as opportunities for reflection and iterative improvement. The module's assessment strategy, comprising a reflective Padlet (an online digital portfolio platform used to organize multimedia reflections) and collaborative table displays, is designed to emphasize the creative process over the final product. This approach aligns with contemporary educational theories that promote experiential learning, constructivism, and creative pedagogies.

The module offers an interdisciplinary education approach that aims to redefine how anatomy is taught and learned. By reintroducing creativity into the curriculum and grounding the module in robust educational theories, the initiative offers a model for how science education could evolve to better engage and inspire students, particularly those interested in biomedical engineering, design innovation, and interdisciplinary applications of anatomy. These theoretical frameworks underpin both the module's design and our interpretation of student experiences.

This article presents a descriptive case study of the SA module, exploring its conceptual development and educational design, and evaluates its impact on student learning through analysis of student feedback. The findings provide novel insight into how the integration of artistic methodologies in anatomy education enhances student engagement, creativity, and interdisciplinary collaboration.

## METHODS

### Module conceptualization and pedagogical framework

**FIGURE 1:**
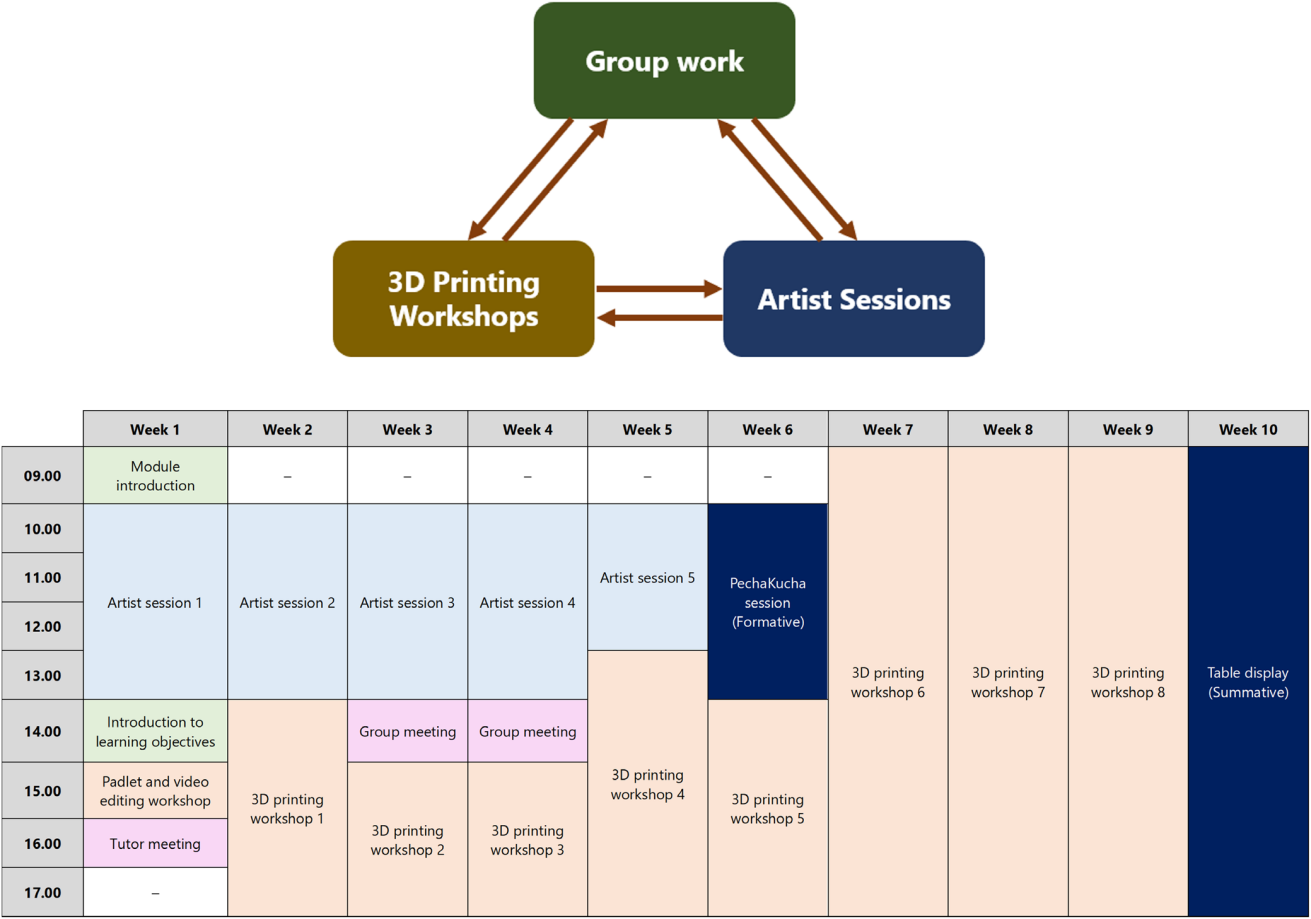
Top: Principal components of the SA module. Bottom: Sample timetable of 22–23 module activities every Friday, across 10 weeks from January to March.

The module, co‐organized by the Department of Anatomy in the School of Bioscience Education and the School of Biomedical Engineering & Imaging Sciences within the Faculty of Life Sciences and Medicine at King's College London, spans 10 weeks from January to March, and is worth 15 credits. All participating students are in their second year of undergraduate study. Since its introduction in 2017, the Synthetic Anatomy module has been delivered annually to second‐year undergraduate students from bioscience and biomedical engineering programs. Cohort sizes have varied across years, ranging from approximately 35 to 73 students between 2018 and 2020 (Table S1). Across this period, module average marks have remained relatively stable (66%–76%), while the proportion of students achieving marks ≥70% has increased in recent cohorts, reaching approximately 73% in 2023/24 and 2024/25. These longitudinal performance metrics indicate sustained student engagement and consistent academic outcomes across multiple iterations of the module. Students are randomly assigned into groups of five to six students, each with a designated tutor, at the beginning of the module. Throughout the the module, students work together to explore anatomical structures within various contexts such as development, evolution, structure and function, brainstorm creative anatomical designs, and bring their ideas to reality with computer modeling and 3D‐printing (Figure 1). This is supplemented by regular in‐person teaching activities every Friday to facilitate their learning, including 3D‐printing workshops and artist sessions. Creative direction of the artist‐led sessions was developed in collaboration with Mr. Les Bicknell from Norwich University of the Arts. His input was central to the tone and conceptual underpinning of the arts‐based sessions and helped establish the imaginative framework of the module.

3D‐printing workshops, led by the biomedical engineering department, provide an opportunity for students to practice using computer‐aided design (CAD) software and 3D‐printing technologies under the guidance and supervision of professors and teaching assistants (biomedical engineering PhD students working as graduate training assistants).

Arts sessions led by visiting artists form a component of the module. These sessions are designed to foster lateral thinking, challenge functional assumptions, and explore alternative modes of anatomical expression. Each activity aims to destabilize the notion of fixed anatomical truth and instead invite students to speculate, hybridize, and personalize structure through imaginative reconstructions. Examples include:

- *Bag Anatomy*: This exercise explores alternative ways of communicating information using senses beyond vision and speech. Students work in pairs with a bag containing concealed objects. One student is allowed to place their hands inside the bag but cannot speak, write, or draw, while the second student cannot touch the bag and must document the information communicated by their partner. Through gestures and tactile exploration, students attempt to convey details such as shape, texture, or size of the hidden objects. The activity highlights how sensory perception and interpretation influence communication and encourages students to consider alternative approaches to conveying anatomical information. It aligns with Kolb's model of active experimentation and Piaget's cognitive constructivism by encouraging learners to question and reconstruct their assumptions about how information can be communicated and understood in constrained settings.
- *Imaginative Observation*: students are given a selection of historic anatomical models and asked to draw an imaginative anatomical model of their own. They are then tasked to design a third model as a hybrid of the previous two, creating an “evolution timeline” from the original historic model to the imaginative model with the hybrid in between. This exercise encourages students to brainstorm how their innovative designs could evolve from current anatomical structures. Students then bring their designs into reality by translating them into 3D cardboard models, which are exhibited as a mock table display. This exercise allows students to develop their spatial awareness and brainstorm ways to effectively utilize the dynamic perspectives of 3D space in their work, as well as re‐explore and reinterpret the use of anatomical models as art pieces but also as functional teaching tools.
- *Alien Anatomy*: students are given a short prompt about a specific living environment and survival features, for example “an agile predator in the desert,” and are tasked to design an imaginative alien animal with anatomical features best suited to surviving in the given environment. Students used a wide range of arts‐based resources, including unconventional materials such as balloons, to create 3D models of their imaginative creatures.
- *Team Building—Six Thinking Hats:* students engage with Edward de Bono's *Six Thinking Hats* framework,^12^ a structured brainstorming method that encourages groups to consider problems from multiple perspectives. Each “hat” represents a different mode of thinking: White (facts and information), Red (feelings and intuition), Black (risks and potential problems), Yellow (benefits and opportunities), Green (creative ideas), and Blue (managing the thinking process). By sequentially adopting these roles, students evaluate project ideas from multiple viewpoints and structure collaborative discussion.
- *Table Play—Genetic Storytelling:* inspired by the concept of genetic mutation, this session introduced students to symbolic visual communication and its potential for storytelling. Students translate abstract concepts into visual compositions by representing words using three or fewer geometric shapes. These shapes are then modified across successive “generations” to explore concepts such as inheritance, mutation, dominant and recessive traits, and environmental adaptation. For example, a shape representing a particular trait may evolve to reflect changes in environmental conditions or survival pressures. The exercise introduces visual storytelling and speculative evolutionary thinking, helping students conceptualize how anatomical features might develop in response to environmental and functional demands.
- *Collecting, Dissecting and Editing*: each group is introduced to a particular region in the classroom for example, doorway, window, cupboard, ceiling corner etc. They observe and document their experiences in that area using techniques beyond photography or text, for example, pencil drawing, paint, collage, rubbing textures, sound etc. These are collated to paint a “picture” to be shared with a paired group in another space. The second pairing presents their work in the space they were working in and discuss whether the presentation accurately brought the experience to life. This motivates students to express their ideas and experiences using non‐traditional methods.

The module design was informed by constructivist, experiential, and social learning principles described in the Introduction. The artist sessions and 3D‐printing workshops were designed to support exploratory hands‐on learning through experimentation, discussion, and iterative design. Collaborative learning was supported through interdisciplinary group work and facilitated teaching sessions in which tutors and graduate teaching assistants provided feedback during project development. Students documented their learning through individual eportfolios structured around Gibbs' reflective cycle. Artist sessions in particular help students construct meaning in anatomy and creative design through imaginative, hands‐on exploration. For example, *Genetic Storytelling* invites students to experiment with symbolic visual communication, translating abstract genetic concepts into 3D artifacts. This encourages exploration of both aesthetic and functional elements of design through iterative prototyping. Similarly, *Imaginative Observation* and *Alien Anatomy* prompt students to create anatomical forms that solve specific challenges or reflect hypothetical evolutionary paths. These sessions promote critical thinking, creativity, and spatial reasoning, helping students consolidate their understanding of evolutionary anatomy through practical experimentation and reflective analysis.

This method of learning is further reinforced by group discussions, where students exchange ideas and constructive criticism, what is termed “social constructivism.” It stems from Vygotsky's theory that students cannot acquire knowledge effectively on a solitary basis as learning is highly dependent upon interactions with others. This involves (1) interactions with peers through group discussions, and (2) interactions with more knowledgeable educators who act as facilitators and provide external guidance. The importance of support from facilitators in active learning is emphasized in Vygotsky's concept of the Zone of Proximal Development (ZPD), concerning the distance between development achieved through independent learning and the potential developmental level that could be obtained with expert guidance. The ZPD can be thought of as a form of “scaffolding,” a construct proposed by Bruner^13^ that is withdrawn as it gradually becomes internalized. In this manner, interactions with facilitators via tutorial and feedback sessions are gradually internalized during the learning process to form mental constructs through the ZPD. The role of a facilitator is not simply to pass on information from teacher to student, but to guide students' self‐directed learning and ensure that it aligns with the intended learning objectives. Throughout the module, social constructivism is fostered through interdisciplinary group work in designing table displays with supervision from tutors, and similarly through artist and 3D‐printing sessions which provide a friendly environment for peers to work with one another with support from experienced artists, graduate teaching assistants and academics who serve as session facilitators.

Halfway into the module, students present their project ideas and developmental progress in the form of a PechaKucha, a presentation format originating from the early 2000s that has gained popularity in group work and education. In its original form, the speaker offers 20 slides, with 20 s to present each slide.^14^ Each slide is comprised of one picture only with no written text, encouraging efficient use of imagery and spoken words to create a memorable and concise presentation in a storytelling manner.

This presentation format was adopted as part of the formative assessment, where each group creates a 1‐min presentation of their project idea using three to four slides. Presentations are delivered in front of the entire cohort, followed by feedback from peers and tutors. The session provides an opportunity for students to gauge the progress of other groups and exchange ideas in an informal, engaging atmosphere aligning with peer learning.^15^ Feedback from tutors aid the alignment of project aims with learning outcomes, clarity of anatomical concepts, and originality of visual storytelling. Students then use this feedback to iteratively refine both the design and narrative structure of their project.

At the end of the module, students' final group projects are showcased via unmanned table displays. Students are required to make effective use of 3D space with a range of props and imagery to create a final table display, guiding the viewer's attention along the various stages of project development, from conceptualization to the finished product, as well as the challenges and failures in between. Table displays are assessed by module tutors and academic staff involved in the module based on creativity and originality of project ideas, artistry of display, and the quality of narrative structure and effectiveness in conveying their learning experiences.

**FIGURE 2:**
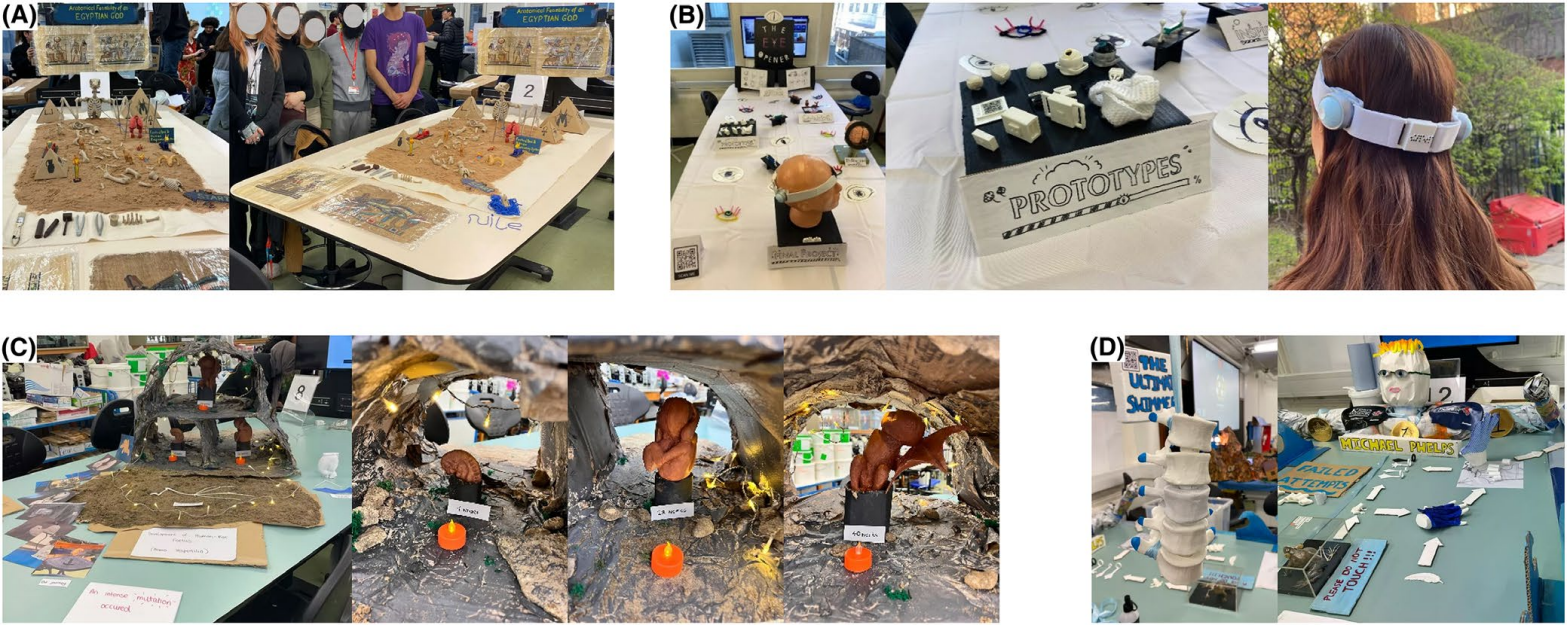
Sample project themes and table displays made by students. (A) Anatomy of an Egyptian god; (B) the “Eye‐opener”; (C) human foetus with bat‐like wings; (D) the ultimate swimmer.

Sample projects presented by students in past years include:

- *Anatomy of an Egyptian God* (Figure 2A): the group was interested in exploring the skeletal anatomy of an ancient Egyptian god, drawing inspiration from the artistic designs of Ra (God of the Sun) and Isis (Goddess of Healing) which incorporated anatomical features of both humans and birds, particularly the integration of avian wings into the human thoracic skeleton. Their display featured a model of an excavation site with a 3D‐printed Egyptian god skeleton of their own design.
- *The “Eye‐opener”* (Figure 2B): the group designed a headband with moveable prosthetic eyes at the back of the head to provide 360° vision, incorporating the anatomy of the orbit into a wearable device. Their display featured a timeline of initial design sketches, prototypes, and failed prints, and the final product.
- *Human Foetus with Bat‐like Wings* (Figure 2C): the group was interested in exploring what the anatomy would look like if a human were able to fly. This led to the idea of integrating the human skeleton with the wings of a bat, the only known flying mammal. Their display featured a model of a bat cave showcasing the embryological development of the bat‐human foetus at 4 weeks, 28 weeks, and 40 weeks.
- *The Ultimate Swimmer* (Figure 2D): skeletal models of a conceptual human adapted to swim most efficiently, taking inspiration from the webbed feet of frogs and the flexible spines of dolphins.

Alongside table displays, each student submits an individual eportfolio documenting their learning experience. This includes photographic documentation and critical evaluation of their project designs, artworks from artist sessions, and 3D‐printing workshops, and obstacles they encountered during teamwork and collaboration.

A pivotal but often unfamiliar element for science students is the introduction of “CRIT,” a structured critique session adapted from art and design education.^16^ This fosters open dialogue, where students are encouraged to present developing ideas, receive constructive feedback, and engage with multiple viewpoints. Rather than focusing solely on outcomes, the CRIT values process, experimentation, and collective inquiry. It cultivates confidence, critical reflection, and the ability to articulate creative intentions. Functioning as a community‐building tool, the CRIT sessions offer a collaborative space in which knowledge is co‐constructed through guided peer and tutor interaction. CRIT sessions are conducted at the end of the unmanned table display exhibition, where students were brought together to evaluate each other's work critically, offer constructive feedback, and use these insights as the foundation for their final individual eportfolio reflections, particularly in considering what they might have done differently after seeing the full range of final displays.

**FIGURE 3:**
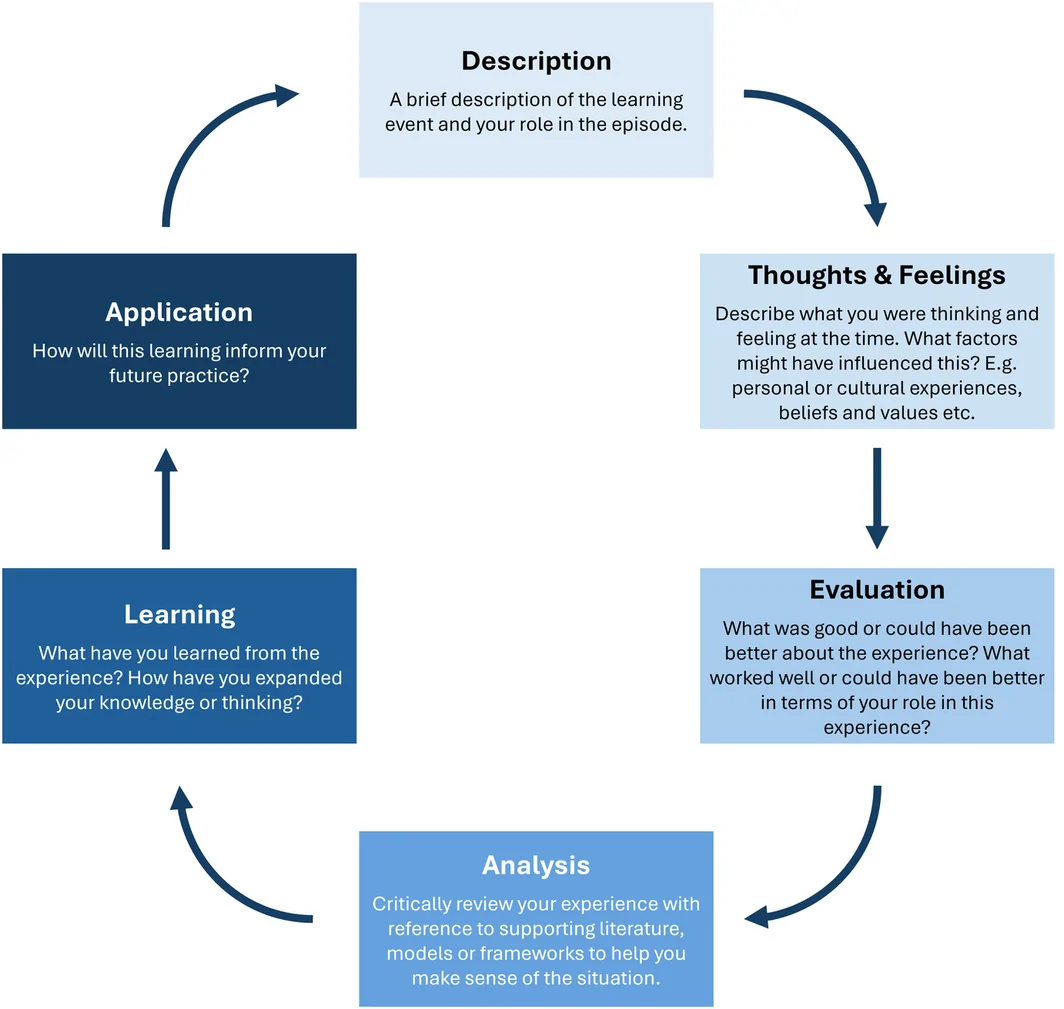
Gibbs' reflective cycle.

The combination of table displays and individual eportfolios constitutes the module's summative assessment, which focuses on reflection of the learning process over the final outcome. Reflection involves critically evaluating one's learning experiences, identifying what was done well, areas that need improvement, and what could be done differently, thereby supporting the development of reflective practice.^17^ It serves as the critical bridge between experiences and new concepts, to enhance and internalize knowledge and understanding of the subject. Reflection is a vital component of Kolb's cycle of experiential learning, encompassing both “reflective observation” (evaluating the experience) and “abstract conceptualization” (learning from the experience and linking new concepts). Throughout the module, students engage in the iterative process of trial‐and‐error as they conceptualize project ideas and design 3D prints. They are therefore encouraged to showcase the entire developmental process, including failed prototypes, within their final table displays. Complementing this, students use their eportfolios to reflect on their learning journey using Gibbs' reflective cycle,^18^ a six‐stage circular framework that guides learners through the process of learning from experience: description, feelings, evaluation, analysis, conclusion, and action plan (Figure 3).

**FIGURE 4:**
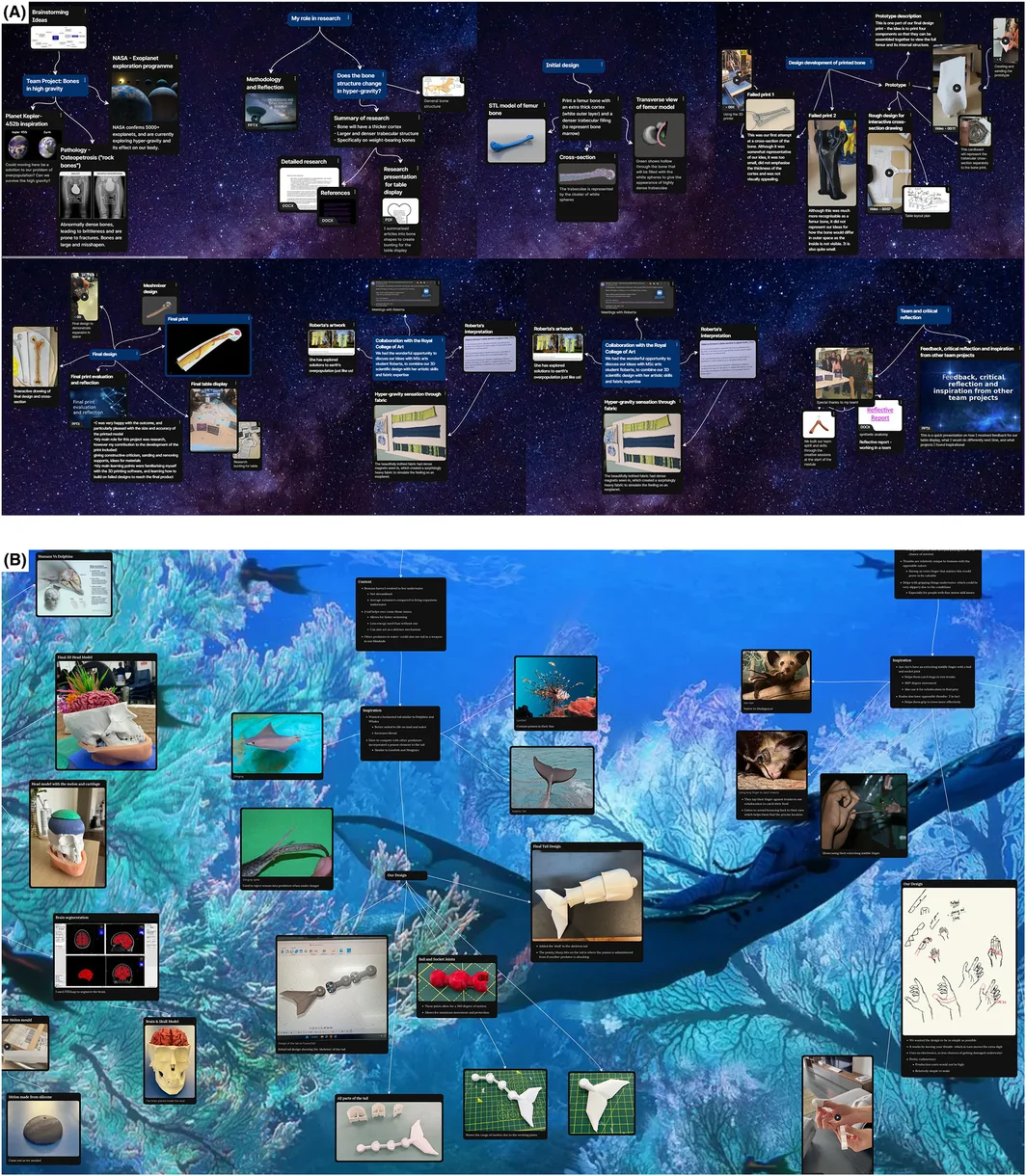
Sample Padlet portfolios from students showcasing their group projects and reflections. (A) Exploring the hypothetical structure of the human femur under hypergravity. (B) Exploring the human anatomy adapted to life underwater, taking inspiration from the unique anatomical features of dolphins and fish.

Historically, eportfolios were assessed using the Mahara ePortfolio System (https://mahara.org/). This was later replaced by Padlet (https://padlet.com/) in 2021. Though functionally similar to Mahara, Padlet offers several unique features. Unlike Mahara where sections are navigated via a drop‐down menu, Padlet provides a “bird's eye” view of the portfolio's overall structure and its components.^19^ This makes the portfolio more visually appealing and easier to follow for the reader, and allows students to ensure that their portfolio is well‐balanced, covering all aspects equally without too much focus on a particular element. Additionally, the absence of a preset structure, along with the drag‐and‐drop style of importing text and multimedia, allows more creative freedom for designing and arranging portfolio contents. Some students created mind maps, while some made timelines (Figure 4). The emphasis on personal reflection via Padlet draws on Kolb's experiential model, where reflective observation is crucial to transforming experiences into conceptual understanding.

The marking rubrics for the table display and reflective work have undergone several revisions to enhance transparency, provide clearer expectations for students, and support more consistent and fair assessment practices across all submissions. Marking of the individual reflective Padlet emphasizes the quality of the narrative structures and their effectiveness in conveying the lessons learned, evidence of research and future solutions, while assessment of the group table display focuses on the novelty and scope of the problem, technical ambition in product design and printing, and artistry of the display. Effectiveness of teamwork is also emphasized, particularly in the utilization of scale and materials, and pride taken in the finished product as a team. This comprehensive assessment approach aims to facilitate a supportive environment where students feel confident to take risks and engage deeply in both the content and the collaborative process.

### Collection and synthesis of student feedback

To evaluate the module's impact on student learning, feedback on the module was collected from students over the years through focus groups, and from tutors through individual semi‐structured interviews. All students enrolled in the module during the study period were eligible to participate in the feedback study. Participation was voluntary, and informed consent was obtained via a consent form prior to inclusion. Participation in focus groups (students) and interviews (tutors) was on an opt‐in basis. Focus groups and interviews used a semi‐structured format, with prompts addressing experiences of creative learning, interdisciplinary collaboration, teamwork, assessment, and access to resources. Qualitative data were analyzed using inductive thematic analysis informed by Braun and Clarke's six‐phase framework: familiarization with the data, generation of initial codes, searching for themes, reviewing themes, defining and naming themes, and producing the final analysis.^20^ The codes extracted included *creative freedom*, *interactive teaching*, *interdisciplinary group work*, *teamwork and communication*, *reflective learning*, *transferable skills*, and *learning resources*. Initial codes were generated inductively from repeated features in the dataset. Numeric identifiers, where used, served only as organizational labels during coding and did not represent analytic ranking or weighting. Related codes were subsequently reviewed and grouped into broader candidate themes through iterative discussion. Two authors independently coded the data and then compared coding decisions. Discrepancies were discussed and resolved through consensus, with refinement of code definitions where necessary. Coding records and theme development notes were retained to support transparency of the analytic process. Cohen's Kappa showed “almost perfect agreement” between raters (*κ* = 0.844 ± 0.031). Analysis was performed using Microsoft Excel.

## RESULTS

This module has contributed to innovative applications linking anatomical learning with creative design. The module was awarded the *Anatomical Society Education and Innovation Award* in 2023.^21^

Feedback was obtained from a total of 89 students from focus groups across the three academic years (2018–2020). 59 students (66.3%) were from the Bioengineering program, while 30 (33.7%) were from the Biosciences program. Tutor feedback was obtained through six semi‐structured interviews. Sample comments from each emergent theme and associated codes are presented in Table 1.

**TABLE 1:** Sample student comments organized by emergent themes and associated codes from thematic analysis (2018–2020 cohorts).

Theme	Code	Sample comments
Creative exploration	Creative freedom	“Encouraged us to think creatively, and think about the details that we don't normally think about” *(Student 29, Bioscience, focus group 2018)* “Nice change from the usual heavily science‐related modules as it allowed us to think more creatively rather than logically” *(Student 68, Bioengineering, focus group 2020)* “Some of the things brought up by the artists and students I had never thought about. I thought the table is just going to be there from one view. I'd never thought about what if people move around, what if people want to touch it, or what if you want to do lots of things with it? I thought it was very useful” *(Student 40, Bioscience, focus group 2020)* “The amount of effort that people have put into their table displays because they can't be there, they want to look at every little detail… everyone's done different things” *(Student 21, Bioscience, focus group 2019)* “A bit too much freedom” *(Student 44, Bioengineering, focus group 2018)* “Rules around the project weren't clearly defined leaving a sense of uncertainty throughout the semester” *(Student 30, Bioengineering, focus group 2020)* “The criteria and format of the eportfolio was a bit unclear which made it harder to do” *(Student 11, Bioscience, focus group 2018)* “Lack of guidance in project aims, criteria and what everyone was looking for led to difficulty in choosing approaches and designs over project” *(Student 22, Bioengineering, focus group 2019)*
Experiential learning	Interactive teaching	“Very hands‐on experience” *(Student 19, Bioscience, focus group 2020)* “Interactive teaching allows you to recall things better” *(Student 64, Bioengineering, focus group 2020)* “TAs were very helpful during the 3D printing sessions responding to student queries with great expertise and consideration” *(Student 85, Bioscience, focus group 2019)*
	Reflective learning	“Emphasis on the journey rather than the final product” *(Tutor 2, interview)* “Puts a positive spin on failure so you come to the next meeting with growth and ideas to improve” *(Student 56, Bioscience, focus group 2020)*
	Transferable skills	“A great way to explore a different side to this degree and the fields I wish to work in” *(Student 35, Bioengineering, focus group 2020)* “An amazing and unique opportunity to learn how to 3D print, as I would not have had the opportunity any other way” *(Student 58, Bioscience, focus group 2018)* “I came out of this module with new skills and a greater appreciation for engineering and anatomy” *(Student 18, Bioengineering, focus group 2018)* “If we go on to make medical devices and other products, design will be a big part of it and (this module) really helped (me) understand that” *(Student 87, Bioengineering, focus group 2019)*
	Learning resources	“(There was a) shortage of SD cards and available printers during the 3D printing sessions, making it hard to actually print things” *(Student 34, Bioengineering, focus group 2020)* “As someone who is not a bioeng student I do not have much of a background in 3D printing… would have loved step‐by‐step guidance in learning software” *(Student 19, Bioscience, focus group 2018)*
Interdisciplinary collaboration	Interdisciplinary group work	“It was interesting to work with a group that was suddenly put together… a lot of different perspectives and skillsets and personalities on the table… we learnt a lot from each other especially from students doing a purely science degree” *(Student 26, Bioscience, focus group 2020)*
	Teamwork and communication	“In terms of projects we can come up with a lot of things as a group rather than just as individuals … I don't think I could have made our table display on my own” *(Student 74, Bioscience, focus group 2019)* “I quite liked the fact that you could share it with your group, so if someone else wanted to add onto it, it was useful” *(Student 23, Bioengineering, focus group 2019)* “Being the general classroom allowed me to meet new people and make friends, something I needed after coronavirus lockdowns restricting social activity” *(Student 15, Bioengineering, focus group 2020)*

### Creative exploration

Students praised the module's creative freedom in exploring imaginative anatomical designs outside the confines of the textbook, and using 3D modeling techniques to bring their ideas to life. The unique unmanned nature of the table displays also motivated them to explore unconventional methods of showcasing their anatomical models, including motion, sound, and touch. Students attributed the majority of their inspirations to the artist sessions, which prompted them to think outside the box. One student commented it “encouraged us to think creatively, and think about the details that we don't normally think about” (student 29, Bioscience, focus group 2018). Another student commented that it was a “nice change from the usual heavily science‐related modules as it allowed us to think more creatively than logically” (student 68, Bioengineering, focus group 2020).

Despite the benefits, many students expressed concerns about the lack of structure in project objectives and assessment criteria. While creative freedom was appreciated, the absence of clear guidance led some to feel uncertain about expectations. Some students particularly found the artist sessions abstract and struggled to find a connection with their project ideas and learning outcomes. These responses suggest that while creativity is highly valued, it requires structured support, echoing constructivist theories that emphasize the need for balance between autonomy and scaffolding.

### Experiential learning

The module's hands‐on approach was highly valued by students, who considered it a unique opportunity to develop practical skills relevant to their future careers. Many students particularly appreciated the chance to engage with new technologies and highlighted the interactive and practical nature of the teaching, especially in workshops and 3D‐printing sessions. These activities allowed students to connect with the material in a manner that diverged from traditional lecture‐based learning, enhancing their understanding of design principles and teamwork. This approach also provided hands‐on skills they deemed pertinent to their professional aspirations. One student particularly noted, “If we go on to make medical devices and other products, design will be a big part of it and (this module) really helped (me) understand that” (student 87, Bioengineering, focus group 2019). Kolb's experiential learning model underpins this approach, emphasizing learning through experience via a cyclical process of active experimentation and reflective observation, with emphasis on the journey rather than the final product (tutor 2, interview). Practical, hands‐on activities like 3D‐printing align well with this model, as they enable students to engage directly with material, reinforcing their understanding through the application of concepts. As one student noted, “Interactive teaching allows you to recall things better,” (student 64, Bioengineering, focus group 2020) reflecting the principles of active learning. Studies have demonstrated that active participation in the learning process enhances retention of information.^22^ By participating in practical tasks, such as 3D‐printing, students take an active role in their learning, developing critical skills like teamwork, problem‐solving, and adaptability.

However, students also highlighted challenges related to resource availability. A shortage of 3D printers and necessary materials hindered their ability to fully participate in these sessions. Logistical issues, including overcrowded classes, competition for resources, and limited technical support, often detracted from an otherwise positive learning experience, leaving some students frustrated. Additionally, students felt that they had not received sufficient guidance on the assessment criteria for table display and Padlets.

### Interdisciplinary collaboration

The module fostered strong teamwork and communication skills, particularly within interdisciplinary groups. Many students emphasized the value of collaborating with peers from different academic backgrounds including bioscience and biomedical engineering, noting that it enhanced their ability to work in diverse teams and exposed them to “a lot of different perspectives and skillsets” (Student 26, Bioscience, focus group 2020). This collaborative environment encouraged teamwork and also allowed students to learn from diverse viewpoints, improving their interpersonal communication. Students frequently highlighted the benefits of working in interdisciplinary groups, with many reporting improvements in their teamwork skills and enjoying the opportunity to build new friendships. However, some students raised concerns about group dynamics, specifically regarding imbalances in contributions, feelings of isolation within teams, and unequal access to resources between teams. The benefits of peer learning highlighted by students demonstrate the impact of Vygotsky's Zone of Proximal Development, where collaborative environments enable knowledge construction through dialogue and support.

**FIGURE 5:**
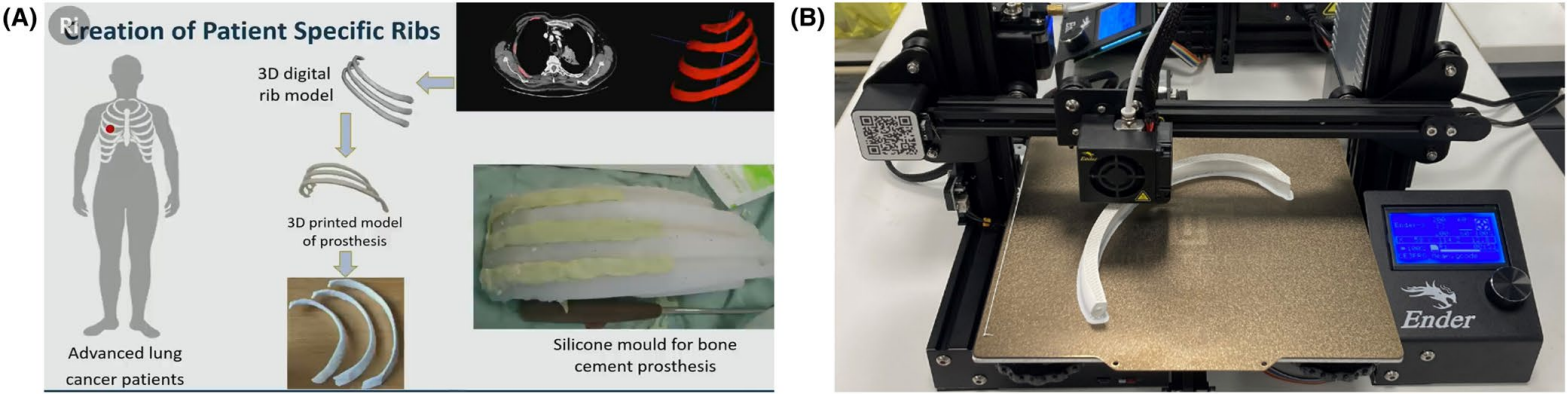
Design of patient‐specific ribs with computer modeling and 3D printing. (A) Design process of synthetic ribs, from computer modeling based on patient CT scans to manufacturing. Image taken from Dr. Pontiki's presentation at the Royal Institution. (B) 3D printing of a prototype model in progress.

The module's impact is further exemplified by the contributions of former biomedical engineering student Dr. Antonia Pontiki from the 2017 cohort. Having developed the idea of using 3D‐printing to create patient‐specific prosthetic ribs for her SA project, she went on to bring her concept into reality for 30 lung cancer patients in collaboration with surgeons from Guy's and St Thomas' Hospitals (Figure 5). Dr. Pontiki's contributions were documented in *Frontiers in Surgery*,^23^ and later on BBC Click and at the Royal Institution.

## DISCUSSION

The SA module created a transformative learning environment that empowered students to explore anatomy through interdisciplinary, creative, and practical engagement. The responses overwhelmingly highlighted how this blend of science and art enabled students to transcend traditional anatomy education, embracing innovative thinking. Underpinning this success are several interlocking educational theories that provide a robust pedagogical framework for interpreting these outcomes. The sustainability of the module is further reflected in consistent academic performance across multiple cohorts, with module averages remaining stable and an increasing proportion of students achieving marks ≥70% in recent years. In addition, the module has expanded substantially since its introduction, growing from cohorts of approximately 35 students in early iterations to around 80–90 students in recent years.

### Creative autonomy and constructivism

One of the most celebrated aspects of the module was the creative autonomy it offered. Students relished the opportunity to ideate and materialize speculative anatomical models, pushing the boundaries of conventional science education. This approach aligns closely with constructivist learning theories, particularly Piaget's cognitive constructivism, which posits that learners build new knowledge through experience and assimilation of new ideas. As students engaged in open‐ended artistic sessions and speculative design, they underwent a process of *disequilibrium*, that is, a mismatch between existing schemas and new challenges, which catalyzed meaningful learning.

However, this same freedom also introduced ambiguity. Some students expressed uncertainty about project goals and assessment expectations. This tension is well documented in constructivist literature; Beghetto^24^ and Bada and Olusegun^25^ emphasize the importance of scaffolding to support learners in navigating creative tasks. To address this, we have introduced more structured guidance and refined rubrics, aligning with Biggs' constructive alignment model, ensuring that intended learning outcomes, teaching activities, and assessments are consistently connected.

### Experiential learning through practice

The module's emphasis on 3D‐printing and hands‐on art sessions exemplifies Kolb's experiential learning theory, particularly the cyclical nature of learning through concrete experience, reflective observation, abstract conceptualization, and active experimentation. Students constructed anatomical models, reflected on their functionality and aesthetics, revised their designs, and implemented improvements, an iterative process central to experiential learning.

This practical engagement also reflects the principles of active learning, which have been shown to significantly improve knowledge retention and skill acquisition.^22^ Students' reflections confirmed this, noting that the hands‐on approach deepened their understanding and allowed them to develop critical, transferable skills such as design thinking, problem‐solving, and communication.

### Social constructivism and peer learning

Collaborative learning was another key strength. The module intentionally formed interdisciplinary teams, promoting diversity in perspectives and problem‐solving approaches^26^ and fostering Vygotsky's social constructivism through peer scaffolding. Theoretical constructs such as the Zone of Proximal Development (ZPD) and Bruner's scaffolding were facilitated through tutor guidance, peer critique sessions, and shared artistic exercises, enabling students to move from dependent to independent thinkers.

The PechaKucha presentations and collaborative table displays offered structured moments of peer interaction and formative assessment, aligning with Topping's theory of peer‐assisted learning.^15^ These activities enhanced cognitive outcomes and also cultivated a community of learning, where students learned as much from each other as from tutors.

However, some challenges emerged around team dynamics, including unequal participation and access to resources. These challenges are consistent with social interdependance theory and cooperative learning literature, which emphasise that effective group work requires intentional design, positive interdependance, individual accountability, and appropriate facilitation.^27^, ^28^. Future iterations of the module will include clearer team role assignments and conflict resolution strategies, grounded in best practices from cooperative learning literature.

### Reflection and lifelong learning

The integration of reflective practice via eportfolios aligns with Gibbs' reflective cycle and Knowles' adult learning theory, emphasizing self‐direction, critical evaluation, and integration of past experiences into new learning. Reflection enables students to derive insights not just from successes but also from failures, turning obstacles into opportunities for growth.

Moreover, the Padlet platform allowed students to express themselves in visually creative and non‐linear ways, reinforcing Bloom's higher‐order thinking skills such as analysis, synthesis, and evaluation, while catering to different learning preferences. This personalized learning approach echoes contemporary calls for student‐centered pedagogies that prioritize flexibility and autonomy.

In summary, the SA module exemplifies a theory‐informed, student‐centered approach to anatomy education. The interplay of constructivist, experiential, social, and reflective learning theories allowed students to engage deeply, take intellectual risks, and develop both scientific and creative competencies. This model enhances anatomy teaching and also provides a compelling framework for interdisciplinary STEM education more broadly.

### Limitations

Although the Synthetic Anatomy module has demonstrated strong educational value, several limitations have emerged throughout its evolution. A primary concern raised by students was the lack of clarity around assessment expectations, which at times led to uncertainty in navigating creative tasks. In response, detailed rubrics have been developed to clearly define performance levels across key criteria, enhancing transparency and consistency in marking. To further support student understanding, a dedicated session on interpreting these rubrics and exploring exemplar projects is planned for the upcoming academic year. Logistical challenges also impacted the student experience, such as limited access to 3D‐printing equipment and materials. To address this, we have expanded the number of printers available and introduced additional time slots to improve access. Another limitation relates to the institutional context in which the module was developed. The Synthetic Anatomy module benefits from access to specialized resources, including 3D‐printing facilities and collaborations with artists and biomedical engineering researchers. Institutions without similar infrastructure or interdisciplinary partnerships may face challenges in implementing comparable activities, which may limit the generalizability of the findings to other educational settings. Concerns around group dynamics, such as unequal participation, were also noted. Moving forward, we are actively encouraging students to see collaborative work as a learning tool and as a reflection of real‐world professional practice, where interdisciplinary teamwork and effective communication are essential. These challenges, and our responsive strategies, represent an ongoing learning process and a commitment to continuous improvement.

## CONCLUSION AND FUTURE DIRECTIONS

The Synthetic Anatomy module exemplifies the potential of interdisciplinary education to reshape how anatomy is taught and experienced. By bridging scientific inquiry with creative exploration, the module fosters a learning environment where students engage deeply with anatomical concepts through hands‐on design, reflective practice, and collaborative problem‐solving. It moves beyond traditional didactic teaching to empower students as creators, innovators, and critical thinkers. While challenges remain, particularly around assessment clarity, resource availability and group dynamics, the responsive changes implemented thus far highlight the module's capacity for growth and refinement. Ultimately, Synthetic Anatomy offers a forward‐thinking model for STEM education that prioritizes creativity, adaptability, and holistic student development. It contributes to this shift by training students to think across boundaries, prototype solutions and collaborate across disciplines, which are vital for the biomedical innovators of tomorrow.

Looking ahead, the module will continue to evolve in response to student feedback and pedagogical reflection. To support sustainability and wider dissemination, a dedicated website has been developed to showcase the module's pedagogical framework and associated projects.^29^ Upcoming changes include the formal integration of assessment literacy sessions focused on rubric interpretation and review of exemplar work, helping students better understand how to align their creative outputs with learning objectives. Efforts to expand 3D‐printing access will also continue, with investments in infrastructure and technical support to accommodate growing student demand. The module will also place increased emphasis on fostering healthy group dynamics through structured collaboration strategies and clearer role assignments. Further measures are being explored to assess long‐term educational impact, such as follow‐up surveys with alumni and integration of digital portfolios into wider career development initiatives. Through these enhancements, the Synthetic Anatomy module aims to remain a dynamic, inclusive, and future‐facing platform for interdisciplinary learning.

## AUTHOR CONTRIBUTIONS

**Mandeep Gill Sagoo:** Conceptualization; investigation; methodology; validation; supervision; data curation; writing – original draft; project administration; resources. **Richard Wingate:** Writing – review and editing; supervision; resources; validation. **Pak Yin Lam:** Writing – original draft; formal analysis; investigation; methodology; validation. **Siham Omar:** Formal analysis; writing – original draft; investigation. **Kawal Rhode:** Writing – review and editing; supervision; resources; validation. **Les Bicknell:** Supervision; writing – review and editing; resources; validation. **Leigh Wilson:** Writing – review and editing; supervision; resources; validation.

## ETHICS STATEMENT

Ethical approval was obtained from the King's Research Ethics Committee in 2018, in compliance with the General Data Protection Regulations (reference no. RESCM‐17/18‐5482). Informed consent was obtained from student participants prior to the study.

## Supporting information


**Supplementary Table 1.** Total number of students who participated in the Synthetic Anatomy module each year.

## Data Availability

The data that support the findings of this study are available from the corresponding author upon reasonable request.
