# Supplementary material for: Integration of the arts and biomedical engineering in innovative interdisciplinary anatomy education: The synthetic anatomy module
Source: Anat Sci Educ. 2026 Jun 11;19(9):1533–45. doi: 10.1002/ase.70285 (PMC13545328; doi:10.1002/ase.70285)
Supplement: Supplementary file 1 [file ASE-19-1533-s001.docx]

Supplementary Table 1. Total number of students who participated in the Synthetic Anatomy module each year.

| **Academic Year** | **Total Student Number** | **Biomedical Engineering** | **Bioscience** |
| --- | --- | --- | --- |
| 2018/19 | 35 | 20 | 15 |
| 2019/20 | 73 | 57 | 16 |
| 2020/21 | 57 | 38 | 19 |
